# Integrative therapy for acute, subacute and chronic facial palsy: repeated differential facial nerve blocks combined with hypodermic needle-based facial nerve stimulation

**DOI:** 10.3389/fneur.2025.1655894

**Published:** 2025-10-29

**Authors:** Hah Yong Mun, So Woon Sirh, Heon Man Sirh, Soo Ji Sirh

**Affiliations:** ^1^Department of Neurosurgery, Gangnam St. Peter's Hospital, Seoul, Republic of Korea; ^2^Department of Anesthesiology and Pain Medicine, Wiltse Memorial Hospital, Suwon-si, Republic of Korea; ^3^Department of Anesthesiology and Pain Medicine, Sirh's Private Pain Clinic, Seoul, Republic of Korea; ^4^Department of Neurosurgery, Sirh's Private Pain Clinic, Seoul, Republic of Korea

**Keywords:** facial paralysis, facial nerve block, facial nerve stimulation, House–Brackmann grading system, Sunnybrook facial grading system, neuroplasticity

## Abstract

**Introduction:**

Despite various recommended treatments, no effective therapy has been established for the satisfactory rehabilitation of subacute and chronic debilitating facial palsy. To address this, we evaluated the safety and efficacy of a novel therapeutic approach that combines repeated differential facial nerve blocks with facial nerve stimulation using a hypodermic needle.

**Methods:**

We retrospectively reviewed 47 patients (acute, *n* = 4; subacute, *n* = 3; chronic, *n* = 40) who were treated at a private pain clinic between January 2017 and December 2023. Patients with persistent facial palsy who were unresponsive to conventional therapies underwent repeated sessions of bilateral facial nerve block following hypodermic needle stimulation of the facial nerves and branches. Facial function was assessed using the House–Brackmann and Sunnybrook grading systems.

**Results:**

More than 85% of patients showed significant improvements in facial symmetry and function. In the chronic group, Sunnybrook scores improved from 42 to 78 (*P* < 0.01), and House-Brackmann grades improved from IV–V to I–II. In the acute and subacute groups, both grading scores showed significant improvement. Transient bruising was noted as a minor adverse event, and the clinical improvement remained stable after treatment.

**Conclusions:**

Our novel integrative treatment is demonstrated to be a safe and effective option for treating intractable subacute and chronic facial palsy.

## 1 Introduction

Bell's palsy, characterized by sudden unilateral facial nerve paresis or paralysis, predominantly has an idiopathic etiology. Several studies have identified herpes simplex virus infection as the principal causative factor of its pathogenesis (1–4). The incidence of Bell's palsy exhibits international variability, but typically ranges from 10 to 40.2 cases per 100,000 individuals annually (1, 2). Facial palsy is typically classified by lesion location (central or peripheral) (5) and temporally as acute (minutes to days), subacute (days to weeks), or chronic (lasting beyond several weeks), although precise definitions are lacking (5, 6).

Although generally self-limiting, approximately 16–30% of patients develop chronic and debilitating sequelae, including persistent paresis, synkinesis (involuntary movements concurrent with voluntary movements), hyperkinesis, oral dysfunction during eating and drinking, speech articulation challenges, facial pain, abnormal lacrimation, contracture, and decreased quality of life (1, 7, 8).

Current therapeutic approaches for Bell's palsy include corticosteroids, antivirals, combination therapy of corticosteroids and antivirals, physiotherapy, botulinum toxin injections, electrostimulation, acupuncture with or without thread lifting, surgical intervention, and photobiomodulation (2, 6, 9).

Nonetheless, major international guidelines, including those from the American Academy of Neurology (10), American Academy of Otolaryngology-Head and Neck Surgery (11), and Canadian medical authorities (1), strongly recommend the administration of oral corticosteroids (prednisone, 50–60 mg per day for 5 days followed by a 5-day taper) within 72 h as the first-line treatment for Bell's palsy (1, 10–13). High-dose corticosteroids (80–200 mg) have also shown favorable outcomes in recent studies (14, 15). Despite various proposed treatments and guidelines for complex facial palsy, no conclusive medical remedy has been established for subacute and chronic palsy (16).

Several studies have indicated that interventions such as botulinum toxin injections with or without thread lifting and selective surgical procedures can mitigate the long-term disability associated with chronic facial palsy (16–21). However, concerns remain regarding their efficacy, safety, and accessibility (1, 10, 11). Treatment guidelines offer no clear recommendations for managing chronic facial palsy, underscoring the need for individualized, multidisciplinary, and multimodal treatments to achieve safe and satisfactory outcomes (16).

Given the heterogeneous progression and multifactorial nature of facial palsy, there is a growing interest in integrative treatments that combine nerve stimulation, modulation, and structural support for optimal personalized results (16). However, evidence-based, comprehensive strategies that effectively address the diverse mechanisms of subacute and chronic facial palsy, including neuromuscular dysfunction and myofascial complications, remain insufficient.

Therefore, we developed a novel integrative treatment method that combined repeated differential facial nerve blocks with hypodermic needle-based stimulation of the facial nerve and its branches (sometimes corresponding to acupoints). This method also incorporates targeted myofascial traction to address fibrosis and contracture without relying on electrical or manual stimulation.

Our approach is based on the hypothesis that repeated facial nerve stimulation and an asymmetrical differential nerve block, which delivers greater anesthetic volume to the unaffected side than to the affected side, may transiently rebalance afferent input to the brain, thereby facilitating neuroplasticity in both the facial nerve and motor cortex. This non-electrical multimodal strategy aims to improve clinical outcomes by enhancing neuromodulation via peripheral and central mechanisms. This method also aims to alleviate myofascial fibrosis and contracture by incorporating targeted facial traction techniques into the injection and stimulation processes.

This retrospective study aimed to evaluate the efficacy, safety, and potential clinical benefits of this integrative treatment method in patients with acute, subacute, and chronic facial palsy who were unresponsive to standard therapies.

## 2 Materials and methods

This study was approved by the Wiltse Memorial Hospital's Joint Research Ethics Committee Institutional Review Board (2024-W13), was granted a waiver of written informed consent owing to its retrospective design, and adhered to the ethical standards of the Declaration of Helsinki.

**Figure 1:**
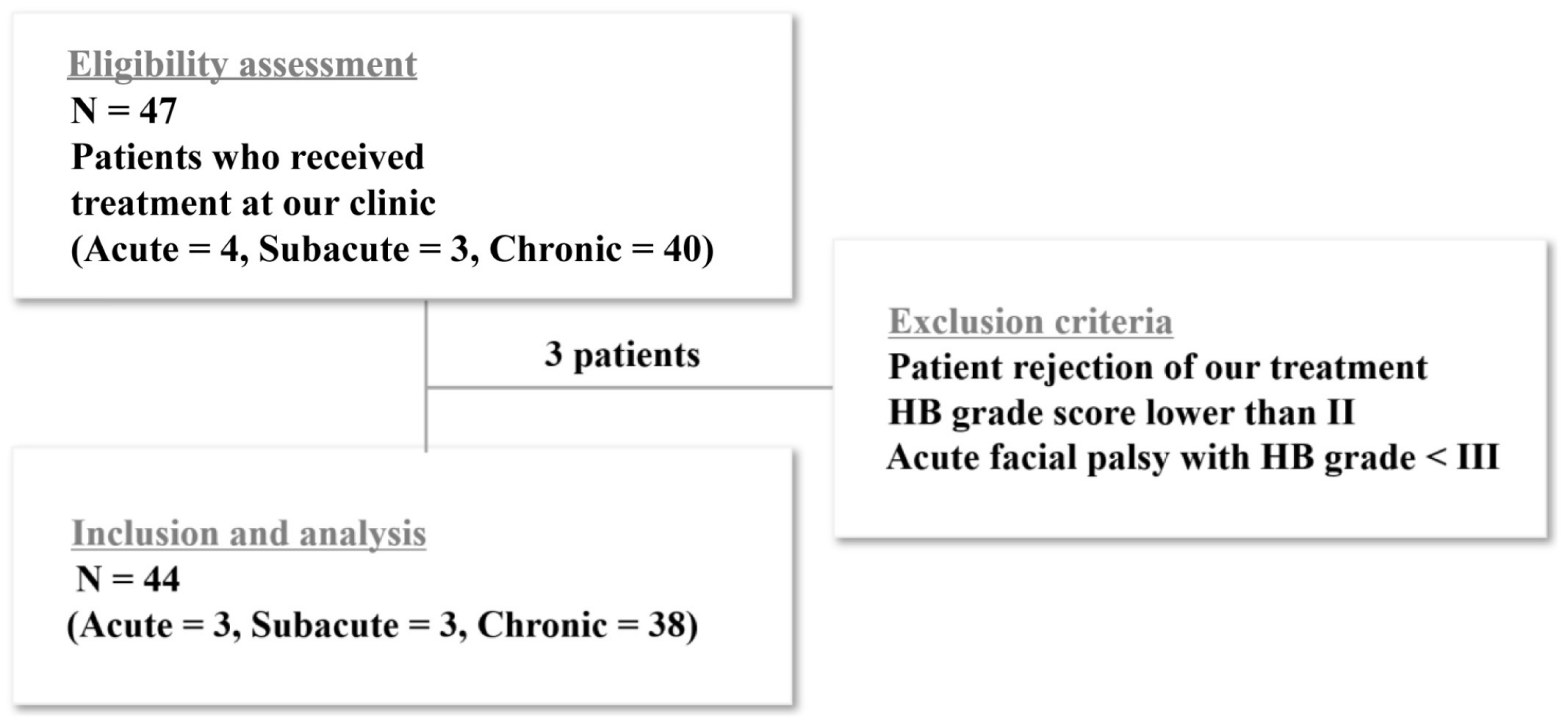
Flow chart of patient enrollment. Among the enrolled patients, 47 met the inclusion criteria and completed the study. Three patients were excluded because they either dropped out of the study or met the exclusion criteria. For the statistical analysis, the acute and subacute groups were combined and compared with the chronic group.

Facial palsy was categorized into acute ( ≤ 3 wk), subacute (3–6 wk), and chronic (>6 wk), according to symptom duration. We reviewed the records of 47 patients treated at Sirh's Private Pain Clinic in Seoul from January 2, 2017, to December 31, 2023. Among them, 44 patients (acute, *n* = 3; subacute, *n* = 3; chronic, *n* = 38) met the inclusion criteria and completed the treatment. Three patients were excluded because they dropped out or met the exclusion criteria (Figure 1).

### 2.1 Inclusion and exclusion criteria

The inclusion criteria were as follows:

1. acute, subacute, and chronic facial palsy or paralysis;

2. consent to undergo integrative treatment;

3. persistent or recurrent symptoms;

4. no satisfactory response to standard therapies including corticosteroids, botulinum toxin, acupuncture, or selective surgical procedures;

5. either unilateral or bilateral presentation.

The exclusion criteria were as follows:

1. Patients who prematurely stopped treatment for any reason;

2. Patients with House–Brackmann (HB) score ≤ II or acute-phase facial palsy with HB grade < III. These cases were excluded because they generally demonstrate favorable outcomes with oral corticosteroids and are often managed during the early stages with acupuncture in Korea;

3. Patients in the acute stage suspected of central facial palsy who present with facial weakness sparing the forehead.

These patients were excluded because of minimal symptom-related distress and limited willingness to undergo our treatment protocol, which is often attributed to a low perceived need for intervention or concerns regarding treatment-related costs and time commitment.

### 2.2 Procedure

#### 2.2.1 Newly designed 30G, 1.9–3.8 cm thin hypodermic needles

**Figure 2:**
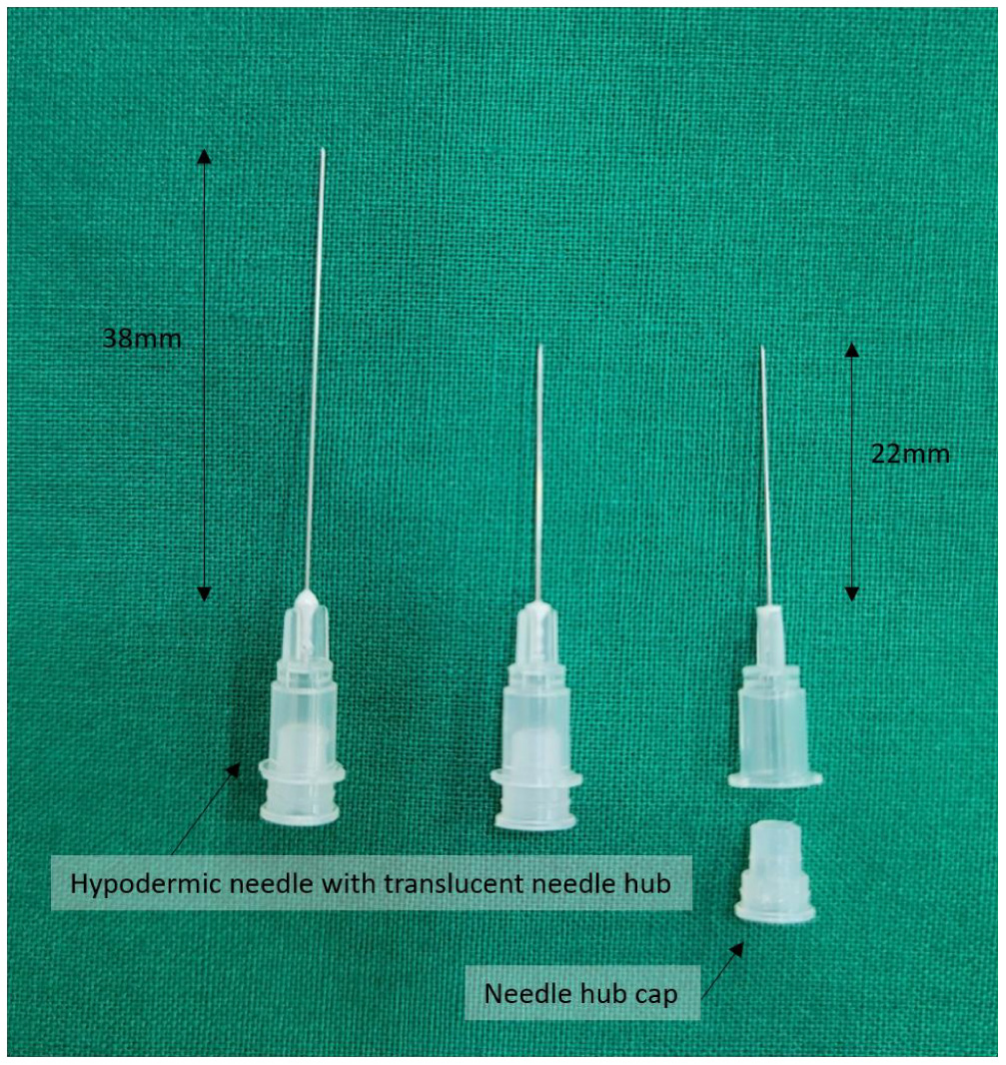
Innovative 30G hypodermic needles (length: 1.9–3.8 cm) with transparent plastic hubs and caps, designed to enhance procedural safety by enabling visual confirmation of blood or cerebrospinal fluid during needle placement. The device was developed by Dr. Hun Man Sirh and has been officially registered as a utility model with the Korean Intellectual Property Office (Registration No. 0399639).

Innovative 30G hypodermic needles (1.9–3.8 cm in length) with transparent plastic hubs and caps (Figure 2) were used in the management of acute, subacute, and chronic intractable facial palsy, as well as associated tinnitus, motor-sensory dysfunction, and pain syndromes refractory to conventional medical, surgical, traditional acupuncture, or neuromodulatory approaches.

These needles are designed to reduce the risk of inadvertent nerve or vascular injury during needle placement or anesthetic administration by allowing direct visual confirmation of blood or cerebrospinal fluid, thereby eliminating the need for aspiration.

The needle design was officially registered with the Korean Intellectual Property Office as a utility model (Registration No. 0399639) and was originally developed by Dr. Hun Man Sirh.

#### 2.2.2 Facial nerve approach

**Figure 3:**
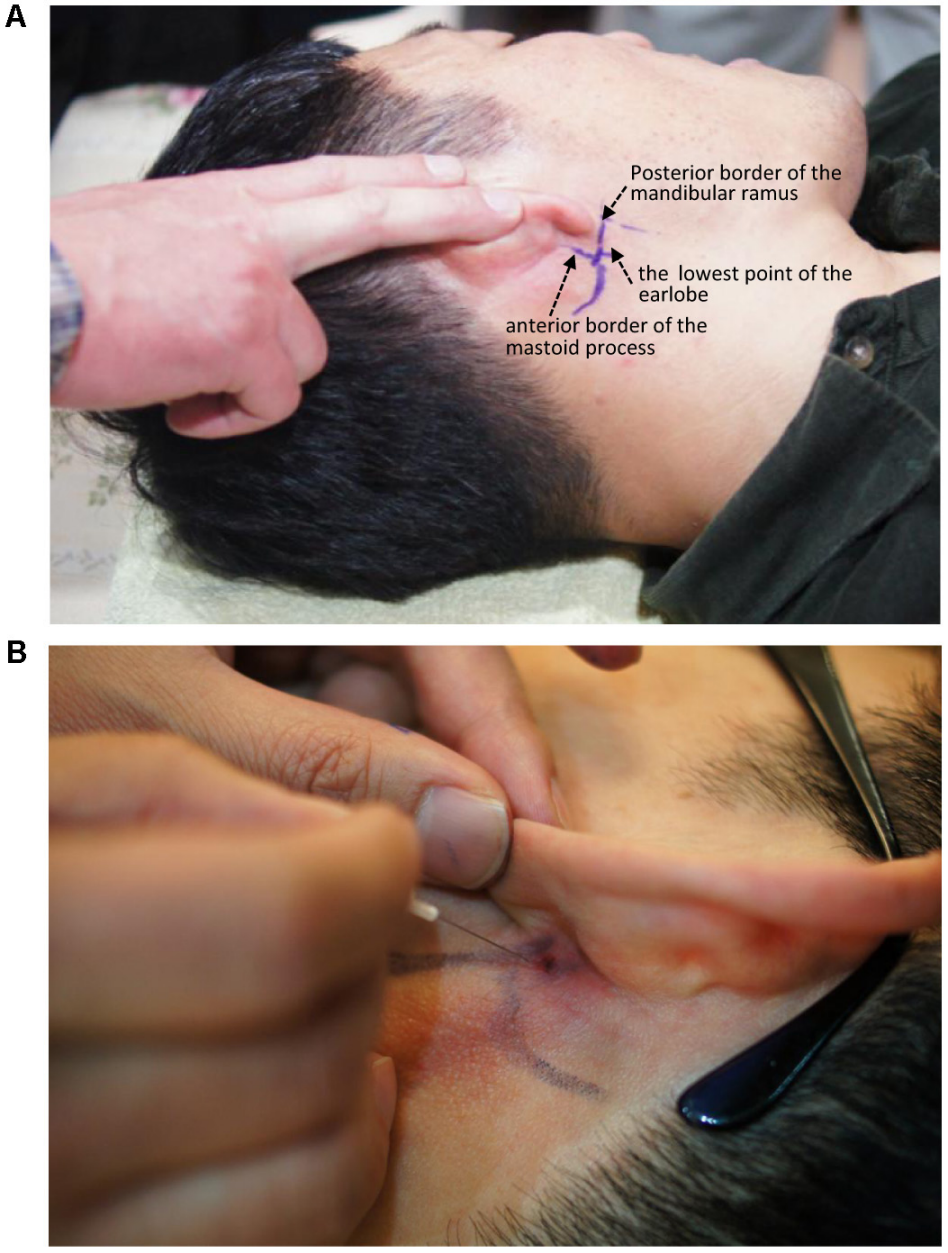
Needle entry point for administering a facial nerve block. **(A)** Entry point. **(B)** Needling direction.

The entry site was the area where the skin was indented upon finger palpation (usually using the fifth fingertip) between the posterior border of the mandibular ramus and the anterior border of the mastoid process, above the lowest point of the earlobe (Figure 3).

#### 2.2.3 Integrative treatment method and course

**Figure 4:**
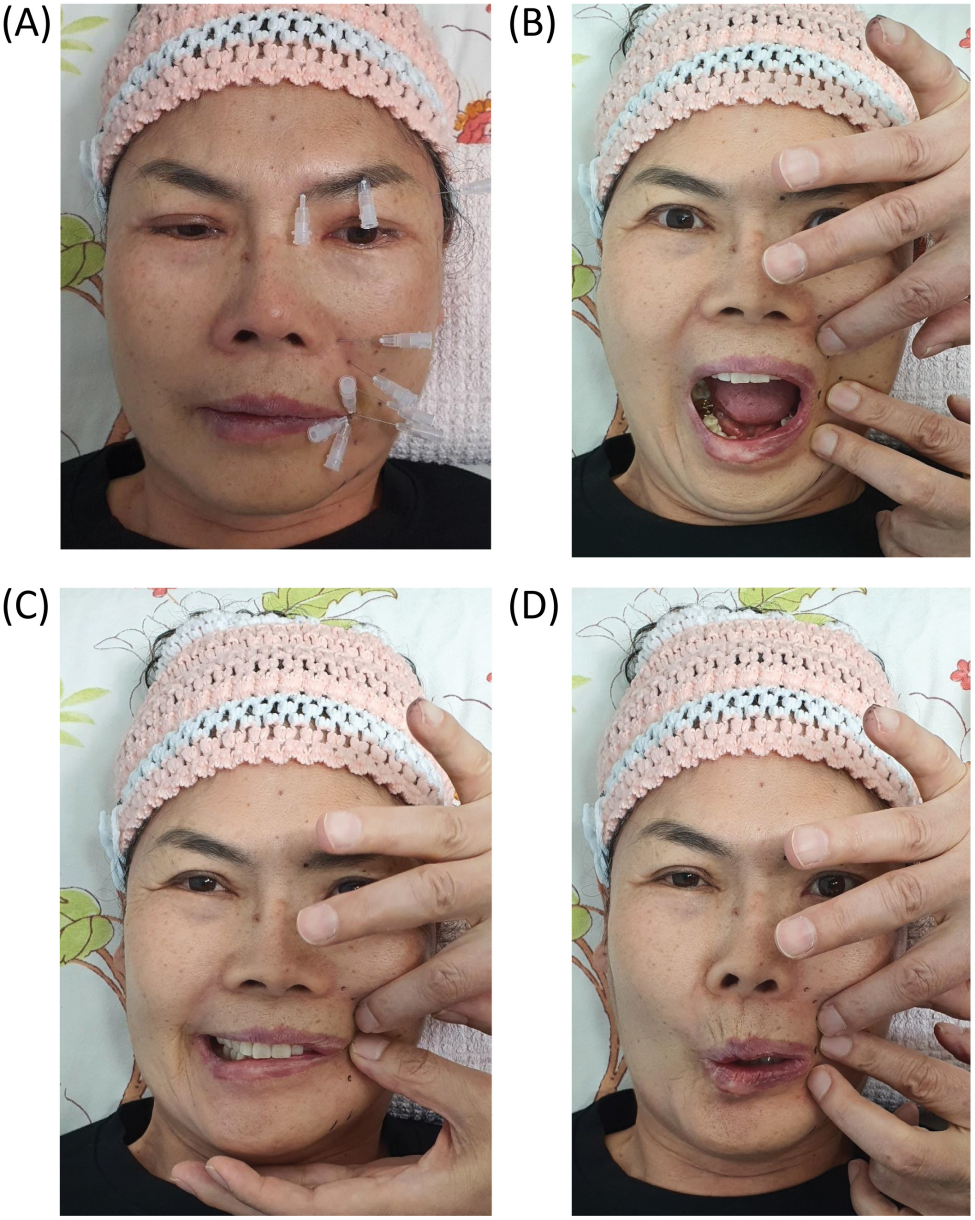
Representative clinical photographs showing key components of the integrative treatment protocol for subacute and chronic facial palsy. **(A)** Hypodermic needle insertion targeting multiple peripheral branches of the facial nerve on the affected hemiface. Needle placement was guided by palpation and anatomical landmarks to deliver localized mechanical stimulation and pharmacologic modulation. **(B–D)** Dynamic assessment of facial symmetry during phoneme articulation (“A,” “E,” and “O”) using finger traction maneuvers to determine the most symmetrical bilateral entry points for intervention. Patient consent was obtained for clinical photography and publication.

All patients who met the inclusion criteria began treatment immediately after providing their informed consent. Each session used a custom 30G hypodermic needle (1.9–3.8 cm) with a transparent plastic hub. The needle was inserted bilaterally near the stylomastoid foramen to target the main trunk of the facial nerve and into the affected facial regions to target symptomatic peripheral branches (Figure 4A).

The therapeutic regimen was tailored to manage facial muscle hyperfunction and weakness by employing finger traction maneuvers (e.g., lifting, pulling, and multidirectional skin tension) for differential facial nerve block and needle steering. The operator identified the most symmetrical point bilaterally using finger traction to manipulate the facial soft tissue. This site was selected as the entry point for needle insertion, and the needle was advanced horizontally along the fascial plane where the facial nerve typically resides (Figures 4B–D).

These techniques are aimed at enhancing facial symmetry, reducing muscular hypercontraction, and attenuating synkinesis. No local anesthesia was employed during the hypodermic needle insertion and stimulation procedures. Mechanical needle stimulation was applied for 20–30 min (acute/subacute) or 30–40 min (chronic). Differential facial nerve block was performed after stimulation. A total of 0.5% lidocaine was administered near the stylomastoid foramen, with 1–2.5 cc injected on the unaffected side and 0.5 cc on the affected side, corresponding to a 1:2 to 1:5 ratio, with higher ratios applied in cases of more severe facial paralysis. In rare cases, a combination of 0.5% lidocaine on the affected side and 1% lidocaine on the unaffected side was administered at a ratio of 1:2 to 1:3. Additional injections of 0.2 to 0.3 cc of 0.5% lidocaine were administered directly to the peripheral branches of the facial nerve on the affected side.

These blocks were conducted after a 20–40-min period of needle-induced nerve stimulation and myofascial traction using a hypodermic needle, which paralleled the initial finger traction maneuver.

Our approach sought to temporarily equalize the afferent nerve signals from both sides of the face. Our differential nerve block involved adjusting the dosage or concentration of the anesthetic injection according to the severity of facial paralysis, as well as the desired duration of temporary paralysis on the unaffected side. The overall procedure typically lasted 50–60 min.

To optimize patient comfort during and after the therapeutic procedure, we employed a carefully controlled method to temporarily suppress normal nerve function on the healthy side for a specified duration (typically 10–30 min). Treatment was initially administered two to three times per week. The frequency and total number of sessions were individualized according to clinical response, particularly improvements in HB and Sunnybrook facial grading system (SFGS) scores, with adjustments ranging from one to three times weekly and a total of approximately 10 to 20 sessions, rather than being based on a fixed protocol.

**Figure 5:**
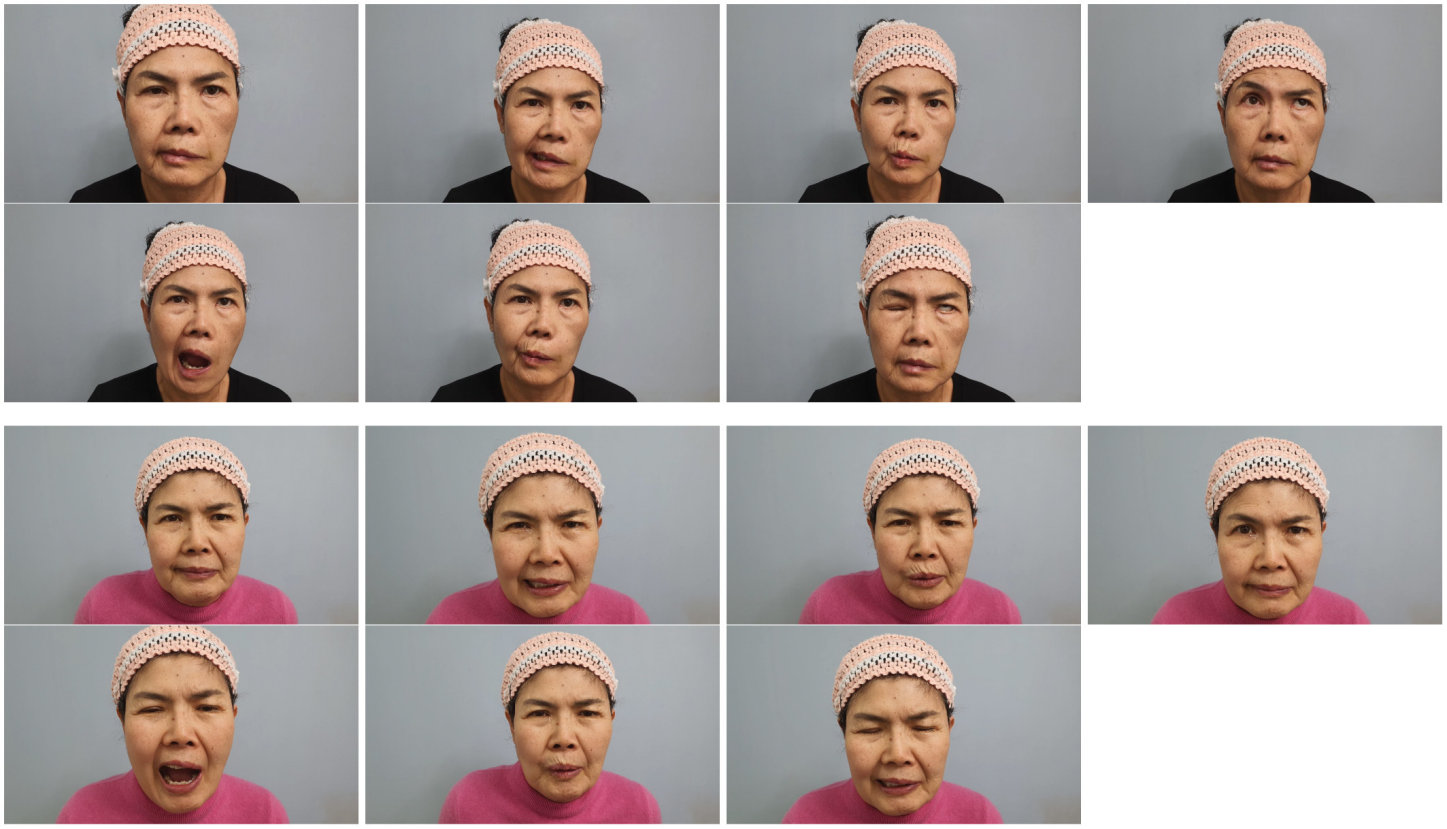
Representative facial expressions before and after integrative treatment in a 64-year-old woman with chronic facial palsy. Frontal photographs were taken at the initial visit (top row) and after 15–20 treatment sessions (bottom row) administered 2–3 times per week at Sirh's Clinic. The images demonstrate a notable improvement in facial symmetry and muscle activation. Expressions include the neutral position, phonation of “A,” “E,” “O,” “U,” eye closed, and forehead elevation. The patient initially presented with House–Brackmann grade V facial palsy approximately 10 wk after onset.

We describe a representative case for illustrative purposes. A 64-year-old woman with no prior medical history experienced a sudden-onset facial palsy on July 23, 2022. She was immediately admitted to a traditional medicine hospital where she received daily acupuncture and massage; however, her symptoms progressively worsened. Subsequently, she was transferred to a university hospital where she underwent high-dose corticosteroid therapy for 3 wk. Brain computed tomography and magnetic resonance imaging (MRI) revealed unremarkable findings. However, electroneurography revealed degeneration of approximately 63% of facial nerves. Despite continued acupuncture and massage therapy during hospitalization, the patient showed minimal clinical improvement. She presented to Sirh's Clinic on October 4, 2022, approximately 10 wk after symptom onset, with HB grade V facial palsy. After 15 sessions of integrative treatment, substantial functional improvement was achieved. Thereafter, the treatment frequency was gradually tapered to once or twice per week. Repetitive sessions were continued until the patient reached a satisfactory level of recovery, at which point treatment was concluded (Figure 5).

### 2.3 Outcome measures

To assess facial motor recovery, we used the HB and SFGS scores (Supplementary Figure 1) as outcome measures to assess the efficacy of our treatment before treatment initiation and at the final treatment session (22). These two scales are frequently used to evaluate facial nerve function, with the SFGS scale offering higher sensitivity to treatment-induced changes. According to Kanerva et al. (23), HB scores can be approximately converted to SFGS scores as follows: HB I ≈ SFGS 100, HB II ≈ SFGS 70–99, HB III ≈ SFGS 43–69, HB IV ≈ SFGS 26–42, HB V ≈ SFGS 13–25, and HB VI ≈ SFGS 0–12. This conversion allowed us to capture subtle improvements more precisely during post-treatment evaluation. In this study, unsatisfactory outcomes were defined as HB scores >3 or equivalent and SFGS scores below a predetermined threshold of 80.

The primary outcome was the degree of functional recovery evaluated by changes in the HB and SFGS scores between baseline and the final session (3–12 mo).

The secondary outcomes included motor synkinesis, autonomic dysfunction (lacrimation and salivation), and muscular contraction at the end of the study.

### 2.4 Statistical analysis

This study aimed to assess the therapeutic outcomes in patients with acute, subacute, and chronic facial palsy, and to discern the differential effects based on the duration of symptoms. Changes in the HB and SFGS scores were compared before and at the end of treatment. Owing to the small number of patients who presented within 6 wk of onset, the acute and subacute groups were combined into a single group for statistical analysis and compared with the chronic group. This limited sample size is primarily attributable to the fact that patients in the acute or subacute phase often experience spontaneous recovery within 1 month and are more likely to meet the exclusion criteria than those in the chronic phase.

Multiple regression analysis was conducted to identify variables that influenced treatment outcomes. The independent variables included age, sex, number of treatment sessions, disease duration (months), affected side (left or right), and steroid treatment history. Statistical analyses were performed using Stata software (version 17.0; StataCorp LLC, College Station, TX, USA). Descriptive analysis, paired sample *t*-tests, and multiple regression analysis were employed to detect significant differences in the HB and SFGS scores between the two groups. Statistical significance was set at *P* < 0.05. Because this was a retrospective study without a control group, treatment outcomes were evaluated using within-subject pre- and post-treatment comparisons. We chose not to employ traditional nerve blocks, high-dose steroid therapy, combined corticosteroid-antiviral regimens, or acupuncture as a control owing to ethical considerations and their limited evidence of efficacy in treating subacute or chronic facial palsy, as indicated by the current literature guidelines (13).

## 3 Results

### 3.1 Clinical outcomes

Satisfactory recovery was defined as an HB score of II or lower and an SFGS score exceeding 80 at treatment completion. All patients (*n* = 44) met the criteria for satisfactory recovery by the final session.

Minor adverse effects have been reported in a small number of patients. Two patients experienced transient injection-site bruising that resolved spontaneously within 48 h. No cases of nausea, dyspepsia, constipation, or allergic reactions were reported.

Secondary outcome improvements included the resolution or significant reduction in motor synkinesis, lacrimation, and facial tightness. These symptoms were observed in 27 of 38 chronic cases at baseline and improved in 21 cases after treatment. All patients with preexisting myofascial stiffness reported subjective improvements in facial relaxation and symmetry.

### 3.2 Demographic characteristics

Demographic data, baseline HB and SFGS scores, and clinical characteristics of the patients are summarized in Tables 1, 2. Patients were grouped based on symptom duration: the acute/subacute group (*n* = 6, ≤ 6 wk) and the chronic group (*n* = 38, >6 wk).

**Table 1:** Patients with acute and subacute facial palsy: demographic data, House–Brackmann and Sunnybrook Facial Grading System scores, and clinical findings.

**Age/Sex Duration^1^, laterality**	**HBS before treatment**	**HBS after treatment**	**SFGS score before treatment**	**SFGS score after treatment**	**Total number of treatments**	**Medical history**	**Co-morbid symptoms**	**Treatment history**	**History of steroid use**	**Degree of improvement at treatment initiation compared to the peak severity of palsy (subjective description)**
56/F 1 wk, Rt.	3	1	61	100	15		Bilateral facial tremor, ear fullness	Medication	O	No improvement
38/M 6 wk, Rt.	6	2	11	96	16	Herpes zoster, 4 d prior R/O Ramsay-Hunt syndrome	Sudden deafness (90% recovered)	Medication	O	30% improvement
60/M 4 wk, Lt.	4	2	37	96	6	Hypertension		Medication, acupuncture	O	N-C
47/F 6 wk, Lt.	3	1	76	100	6			Medication, manual therapy	O	80% improvement
27/M 10 d, Lt.	3	1	35	100	13			Medication	O	No improvement
55/F 11 d, Rt.	3	1	67	100	17		Eye irritation and tearing, rash around the ear. Complete resolution of tearing after 9 treatments.	Medication	O	No improvement

HBS, House–Brackmann score; SFGS, Sunnybrook Facial Grading System score; N-C, not-checked; R/O, rule out.

^1^The treatment duration was computed starting from the first day of their visit to our clinic.

**Table 2:** Patients with chronic facial palsy: demographic data, House–Brackmann and Sunnybrook Facial Grading System scores, and clinical findings.

**Age/Sex Duration^1^, laterality**	**HBS before treatment**	**HBS after treatment**	**SFGS before treatment**	**SFGS after treatment**	**Total number of treatments**	**Medical history**	**Co-morbid symptoms**	**Treatment history**	**History of steroid use**	**Degree of improvement at treatment initiation compared to the peak severity of palsy (subjective description)**
65/M 3 mo, Lt.	5	2	13	92	30	Liver cirrhosis		Acupuncture		N-C
59/F 4 y, Rt.	4	2	31	92	10		Otalgia	Medication, acupuncture, Botox injection	O	50% improvement after acupuncture, Botox treatment
36/F 5 y, Rt.	3	1	59	100	20		Facial tremor	Acupuncture		N-C
49/F 10 y, Lt.	4	2	31	96	12			Acupuncture		50% improvement after acupuncture
60/F 8 y, Rt.	4	2	22	90	12		Dizziness, fibrotic change of facial muscles	Medication, Botox, decompressive craniotomy operation, ptosis operation	O	N-C
66/F 1.5 y, Lt.	4	2	24	86	12		Synkinesis (eye), periorbital pain	Medication, acupuncture		60% improvement
65/F 2.5 y, Lt.	3	2	56	91	19		Synkinesis, tearing	Acupuncture, stem cell therapy	O	50% improvement
70/M 1.5 y, Rt.	4	2	22	99	17			Medication, acupuncture	O	N-C
64/M 8 y, Lt.	4	2	22	91	3			Medication		N-C
56/M 6 mo, Rt.	4	2	26	90	12	Herpes zoster, 10 d prior, R/O Ramsay-Hunt Syndrome		Medication	O	N-C
43/M 60 mo, Rt.	3	2	55	95	17		Right side facial spasm, synkinesis, ptosis	Medication, acupuncture	O	N-C
43/M 3 y, Rt.	3	2	43	96	13		Hypesthesia on the right V2 area and neck, Fibrotic change of facial muscles Complete recovery of sensation after treatment	Acupuncture		N-C
58/F 1.8 y, Rt.	3	2	57	96	3			Medication, acupuncture	O	N-C
43/M 3 mo, Lt.	3	1	69	100	9		Left-sided facial spasm	Medication, acupuncture		N-C
53/M 5 y, Lt.	3	2	56	95	15	Microvascular decompression operation, 2 y prior	Left side facial spasm, ptosis (aggravated with walking)	Medication	O	N-C
46/M 3 y, Rt.	3	1	57	100	4		Tearing and rhinorrhea during eating	Acupuncture, physical therapy, manual therapy		50% improvement
30/F 2 y, Rt.	3	1	77	100	15	Zygoma fracture operation, 2 y prior	Synkinesis, otalgia	Acupuncture		No improvement
32/M 4 mo, Lt.	4	2	29	91	6	Lt. facial palsy at 13-year-old		Acupuncture		50% improvement
55/M 2 y, Rt.	4	2	35	86	7	Craniotomy, vestibular schwannoma operation	Tearing while eating	Acupuncture, physical therapy, manual therapy	O	30% improvement
50/M 10 y, Rt.	5	2	17	70	35		Synkinesis, fibrotic change of facial muscles	Medication, acupuncture	O	N-C
60/F 3 y, Lt.	4	2	27	86	10		Left-sided facial spasm, facial pain	Medication, acupuncture		50% improvement
45/M 2 y, Rt.	4	1	36	100	8		Left-sided tinnitus	Medication		N/C
58/M 8 y, Lt.	4	2	33	86	10		Drooling, synkinesis	Medication, ptosis operation	O	N-C
69/M 4 mo, Lt.	5	2	17	91	23		Fibrotic change of facial muscles	Medication, acupuncture	O	30% improvement
48/M 10 mo, Lt.	3	2	44	95	8			Acupuncture		60% improvement
74/F 1.8 y, Rt.	3	2	46	96	7	Vestibular neuritis	Dizziness	Medication, rehabilitation training	O	50% improvement
50/M 4 y, Lt.	3	1	49	100	7	Vestibular schwannoma, Lt.	Left-sided deafness, facial tremor, synkinesis	Medication, tumor removal operation, Botox injection	O	40% improvement
57/M 3 y, Rt.	4	2	39	82	30	Herpes zoster	Facial tremor, bilateral hearing difficulty, fibrotic change of facial muscles	Medication	O	60% improvement
43/F 2 y, Rt.	3	1	48	100	20		Synkinesis, hypesthesia of lips	Medication	O	70% improvement
47/M 10 y, Lt.	3	2	42	95	15			Acupuncture		30% improvement
18/M 4 mo, Rt.	3	1	63	100	6			Medication, acupuncture	O	50% improvement
38/M 8 mo, Rt.	4	2	40	90	58		Tearing, tongue numbness, Synkinesis 98% degeneration ratio on ENoG	Medication, stellate ganglion block, physiotherapy	O	N-C
65/M 9 mo, Lt.	3	1	75	100	15	Lt. facial palsy, 30 y prior	Pain on posterior auricular area, left side facial numbness	Medication	O	30% improvement
42/F 2 y, Rt.	4	1	36	100	7		Masticatory discomfort, Eye irritation	Medication	O	N-C
41/M 1 y, Rt.	3	1	74	100	16		Synkinesis (complete recovery after the 7th treatment)	Medication, acupuncture	O	N-C
24/M 9 mo, Lt.	3	1	44	100	18		Synkinesis (complete recovery after the 8th treatment), masticatory discomfort, tearing, dysesthesia on the V2 area	Medication, Botox injection (Masseter muscle)	O	N-C
64/F 2.5 mo, Lt.	5	2	17	88	30		Synkinesis, facial discomfort 63% degeneration ratio on ENoG	Medication, acupuncture, manual therapy	O	N-C
36/M 2 mo, Lt.	3	1	45	100	22		Altered taste sensation, facial discomfort 95% degeneration ratio on ENoG	Medication, acupuncture, physiotherapy	O	N-C

HBS, House–Brackmann score; SFGS, Sunnybrook Facial Grading System score; ENoG, electroneurography; N-C, Not checked.

^1^The treatment duration was computed starting from the first day of their visit to our clinic.

The mean age of the participants was 50.1 y (SD = 13.3; range: 18–74). The study cohort consisted of 28 male and 16 female patients. Facial palsy affected the left and right sides in 21 and 23 patients, respectively. The mean number of treatment sessions was 14.9 (SD = 10.1; range: 3–58). The average duration of facial palsy at baseline was 31.3 mo (SD = 35.5; range: 0.25–120 mo).

### 3.3 Treatment effects

The treatment outcomes are shown in Tables 1, 2. In the acute/subacute group, the mean HB score improved from 3.67 to 1.34 (*P* < 0.001), and the SFGS score increased from 47.83 to 98.67 (*P* < 0.001). In the chronic group, the HB score improved from 3.53 to 1.66 (*P* < 0.001), and the SFGS grading system score increased from 41.47 to 93.82 (*P* < 0.001). There was no statistically significant difference between the groups in the degree of change from pre- to post-treatment in either the HB or SFGS scores.

None of the patients required a surgical intervention. After treatment, in the acute/subacute group, four patients achieved complete recovery with an HB score of I, and two patients achieved an HB score of II. In the chronic group, 13 patients achieved HB score I, and 25 patients recovered to HB score II.

### 3.4 Regression analysis

Multiple linear regression analysis was performed to investigate predictors of treatment outcomes, with the final HB and SFGS scores as dependent variables. The analysis examined the effects of age, sex, number of treatment sessions, disease duration (months), laterality of symptoms (left/right), history of previous steroid use, past medical history, and presence of accompanying symptoms (e.g., synkinesis and contracture). Table 3 presents the results of the multiple regression analyses.

**Table 3:** Multiple regression results.

**Model**	**(1) Dependent variable: Sunnybrook facial grading scores (SFGS)**	**(2) Dependent variable: House–Brackmann scores (HB)**
Age	−0.177 (−2.56)^**^	0.020 (3.90)^***^
Sex	1.583 (0.84)	−0.152 (−1.11)
Number of treatment sessions	−0.231 (−2.67)^**^	0.011 (1.80)^*^
Duration of facial palsy (mo)	−0.068 (−2.75)^***^	0.004 (2.04)^**^
Laterality of symptom	−0.170 (−0.09)	−0.069 (−0.53)
History of steroid usage	0.012 (0.01)	−0.041 (−0.30)
Past medical history (present or absent)	−0.624 (−0.33)	0.021 (0.15)
Accompanying symptoms	−0.547 (−0.27)	−0.295 (−2.00)^*^
Cons	107.441 (24.53)^***^	0.816 (2.55)^**^
F-stats	3.33^***^	4.18^***^
Adjusted *R^2^*	0.303	0.372
*N*	44	44

Corresponding t-statistics are reported in parentheses. ^***^, ^**^, and ^*^ represent significance at 1%, 5%, and 10% levels, respectively.

All models were statistically significant (*F*-statistics, *P* < 0.01) with adjusted *R*^2^ values of 0.303 (SFGS score) and 0.372 (HB score), indicating that the models adequately explained a substantial portion of the variance in treatment outcomes. Multicollinearity was not detected (Variance Inflation Factor < 2 for all variables).

Significant predictors were as follows:

- Age: Older age was associated with poorer SFGS scores (β = −0.177, *P* < 0.05).
- Number of treatment sessions: More sessions were associated with lower SFGS scores (β = −0.231, *P* < 0.05), indicating slower recovery in these patients.
- Duration of facial palsy (months): Longer disease duration negatively affected both SFGS (β = −0.068, *P* < 0.01) and HB scores (β = 0.004, *P* < 0.05).
- Accompanying symptoms: Presence of synkinesis or other symptoms correlated with higher HB scores (β = −0.295, *P* < 0.05).

These findings underscore the importance of early intervention and suggest that age and chronicity are critical prognostic factors.

## 4 Discussion

This retrospective analysis demonstrated that our integrative treatment, which combined hypodermic needle-induced facial nerve stimulation with modified bilateral facial nerve blocks, resulted in substantial functional recovery in patients with acute/subacute and chronic facial palsy who were unresponsive to conventional medical, surgical, or acupuncture-based interventions. Most patients in the cohort achieved satisfactory recovery, supported by statistically significant improvements in both HB and SFGS scores. Regression analysis further identified younger age, shorter disease duration, and fewer required treatment sessions as favorable prognostic indicators, underscoring the importance of early intervention following onset; treatment-resistant facial nerve maladaptive neuroplasticity generally emerges 1.5–2 mo or more after onset (24–27). These findings emphasize that early interventions are critical for optimal recovery. Delayed treatment may allow the establishment of maladaptive neuroplasticity and fixed synkinetic or fibrotic changes, which can affect the facial and amygdalo-motor systems, including facial nerve degeneration and psychoemotional pathology. These compensatory processes are particularly likely to emerge beyond the 1.5-mo post-onset window (24–27). Our data support the notion that prompt initiation of neuromodulatory therapy before the entrenchment of chronic patterns may improve prognosis and reduce the need for adjunctive interventions.

Additionally, meaningful improvements were noted in secondary outcomes such as motor synkinesis and myofascial symptoms, even in chronic-stage patients, despite the lack of statistical testing for these variables. Functional recovery is typically achieved after 10–20 treatment sessions, and early responses strongly correlate with long-term outcomes. Notably, even in chronic cases, sustained improvements in facial relaxation and reductions in synkinetic movements have been reported. Transient complete or significant improvements observed in initial treatments often lead to excellent outcomes. However, the optimal minimum number of treatments required remains unknown. These results highlight the need for a comprehensive investigation into advanced therapies, including integrative needle treatments, electrical or magnetic neuromodulation, and neurostimulation.

We hypothesized that our method would promote functional and mechanical rebalance through both peripheral and central mechanisms. The facial nerve consists of both motor (efferent) and sensory (afferent) roots, including the nervus intermedius, which connects to the autonomic (sympathetic via interconnections and parasympathetic) nervous system. Somatic sensory pathways play a crucial role in the treatment of facial palsy (28). By stimulating both the trunk and peripheral branches (related acupoints) of the facial nerve using fine hypodermic needles in a controlled, directional manner, and by adjusting pharmacologic denervation asymmetrically, our treatment appears to modulate afferent input to cortical and subcortical motor regions. This is likely involved in adaptive neuroplastic processes, as observed in other neurorehabilitation models.

We further posit that repeated needle stimulation induces changes within the facial motor nucleus, corticobulbar pathways, amygdalo-motor circuits, supplementary motor areas, and networks involved in both voluntary and emotional facial expressions. These expressions are generated through the coordinated activity of brain regions, including the amygdala and cortical and subcortical motor areas, which integrate sensory and motor signals to modulate facial output (29). This dual modulation may explain not only the improvements in facial symmetry and voluntary motor control but also the normalization of autonomic symptoms such as lacrimation and salivation. Although our clinical findings suggest improvements in both voluntary and autonomic facial functions, these effects may reflect a broader modulation of facial affect circuits. However, in the absence of direct neurophysiological or imaging evidence, the proposed central mechanisms remain unclear and require further investigations. Furthermore, we hypothesized that needle-guided mechanical stimulation, when applied along the anatomical fascial plane and aligned with the physiological trajectory of the nerve, could reduce aberrant axonal misdirection. In chronic facial palsy with synkinesis, transversely aligned needling may serve as a nondestructive strategy to facilitate accurate axonal reinnervation and prevent maladaptive sprouting. We observed repeated clinical improvements in facial synkinesis cases with fascia-aligned needle retention, potentially mediated by Schwann cell orientation and regeneration guidance (30).

Although various treatments exist for Bell's palsy, current clinical guidelines predominantly recommend high-dose corticosteroids, either alone or in combination with antiviral agents, as the first-line therapy for Bell's palsy (1, 10, 11). However, no consensus exists for treatment beyond the acute phase. Although botulinum toxin, electrostimulation, acupuncture, and selective surgery have been explored, their efficacy, access, and safety remain limited. Studies on electro-acupuncture and manual acupuncture suggest mixed efficacies, and high-quality trials are lacking (31–39). Furthermore, these techniques rarely account for the myofascial or fascial adhesion components in chronic dysfunction.

Conversely, evidence from vagus nerve stimulation (40–44) and contralateral botulinum toxin studies (29, 45) shows that asymmetric modulation of afferent input can enhance cortical adaptive plasticity and recovery. Vagus nerve stimulation-dependent plasticity in the corticospinal, corticorubral, and propriospinal networks likely drives functional recovery improvements (40–44). Similarly, we believe that our treatment, with or without facial motor training, operates through a comparable mechanism, with the added advantages of anatomical precision and non-electrical application. Therefore, it may serve as a safer and more targeted alternative for modulating facial motor plasticity.

Unlike conventional methods, our approach is purely mechanical and pharmacological, and avoids electrical or thermal nerve injury. It incorporates several novel features: (1) bilateral, asymmetrical differential nerve blocks; (2) needle-based peripheral nerve stimulation without electrical current; (3) dynamic myofascial traction and tissue steering guided by palpation; and (4) targeting of both motor and autonomic fibers of the facial nerve. This method was designed to be safe, even for patients on anticoagulants, resulting in minimal complications. Several cases of mild complications have been reported. Some patients experienced transient discomfort during mouth opening, likely owing to a temporary muscle imbalance following a facial nerve block near the stylomastoid foramen. Post-injection pain lasting 1–2 days, along with minimal bleeding or bruising at the needle insertion site, was also observed. To minimize such complications, we consistently used 0.5% lidocaine instead of higher concentrations or long-acting anesthetics.

Myofascial traction, a key component of our protocol, may relieve fascial restriction, reduce synkinesis, and allow more precise needle steering into appropriate nerve planes. This combination of mechanical, pharmacological, and neurofunctional targeting sets our technique apart from existing modalities.

Despite these promising results, further validation is required. Large-scale randomized controlled trials are needed to compare the efficacies of botulinum toxin, electrostimulation, and manual acupuncture. Our treatment approach, which avoids electrical or mechanical damage to the cranial nerves, notably enhances the effects of therapeutic stimulation on the peripheral and central nervous systems, as well as nerve block in chronic and subacute facial palsy cases. Electrical or manual stimulation near large nerves poses risks. To date, no studies have specifically investigated the effects of innocuous mechanical nerve stimulation using needle placement without electrical or manual intervention. Given that recovery from facial palsy appears to be influenced by the duration of needle placement, the therapeutic mechanism warrants further exploration. These outcomes suggest the need for extensive research on integrative needle treatments. Finally, as this treatment involves both peripheral and central systems, future studies should explore its application in other cranial neuropathies and functional somatic disorders involving autonomic dysregulation.

### 4.1 Limitations

This study had certain limitations. As this was a retrospective chart review, randomization and blinding were not performed. Most patients have previously experienced suboptimal outcomes from high-dose corticosteroids, botulinum toxin injections, surgery, acupuncture, or a combination of conventional and traditional therapies. Owing to their psychological distress and refusal to accept randomization, conducting controlled trials with a placebo or sham arm is ethically and practically unfeasible.

Neurophysiological tests, such as electromyography and nerve conduction velocity studies, were not conducted, limiting the objective quantification of nerve recovery. Although composite scores from both HB and SFGS have been used, they are semi-subjective and may not fully capture subtle clinical changes. Although an approximate correlation exists between the two, contemporary research suggests the need for a more precise and standardized facial grading system (23).

Imaging, including brain MRI, was not routinely performed in all patients, particularly those without suspected central lesions. Therefore, only a small number of patients with possible brain lesions were included. Although routine imaging is not universally recommended for idiopathic facial palsy (1, 6, 10, 46), the selective use of MRI and cerebrospinal fluid analysis may be important in cases with suspected otogenic or neoplastic etiologies (46, 47).

Another limitation was the relatively small sample size, particularly in the acute and subacute groups, which restricts the generalizability of the statistical conclusions. Additionally, the cost of treatment was the responsibility of the patients, supplemented in part by health insurance. Future research should include larger prospective randomized controlled trials focusing specifically on repeated facial nerve blocks or needle-based facial nerve stimulation. Objective neurophysiological outcome measures and imaging-based validation will help establish the efficacy and mechanisms of this novel integrative approach.

## 5 Conclusions

Our findings demonstrate that repeated hypodermic needle-guided facial nerve stimulation and differential facial nerve blocks effectively mitigate the persistent sequelae of facial palsy, including facial asymmetry, muscular hypercontraction, synkinesis, and hyperkinesis. This therapeutic strategy represents a novel and promising option for managing subacute and chronic facial palsy, with the potential to guide future research on facial nerve rehabilitation and motor neuron recovery.

Although the subacute group included a limited number of patients, the consistency of improvements across the entire cohort supports the potential clinical utility of this approach. Further studies with larger and more balanced samples are required to validate these findings. Moreover, this strategy could serve as a foundation for future integrative interventions aimed at promoting neurofunctional recovery and neuromuscular rebalancing in complex treatment-resistant facial nerve disorders. Its therapeutic effects may be further enhanced by combining this approach with regenerative techniques such as nerve conduits, stem cell therapy, neurotrophic factor delivery, and other emerging therapeutics.

## Data Availability

The original contributions presented in the study are included in the article/Supplementary material, further inquiries can be directed to the corresponding author.
