# Supplementary material for: Integrative therapy for acute, subacute and chronic facial palsy: repeated differential facial nerve blocks combined with hypodermic needle-based facial nerve stimulation
Source: Front Neurol. 2025 Oct 29;16:1655894. doi: 10.3389/fneur.2025.1655894 (PMC12605347; doi:10.3389/fneur.2025.1655894)
Supplement: Supplementary file 1 [file Table_1.docx]

Supplementary Material

**
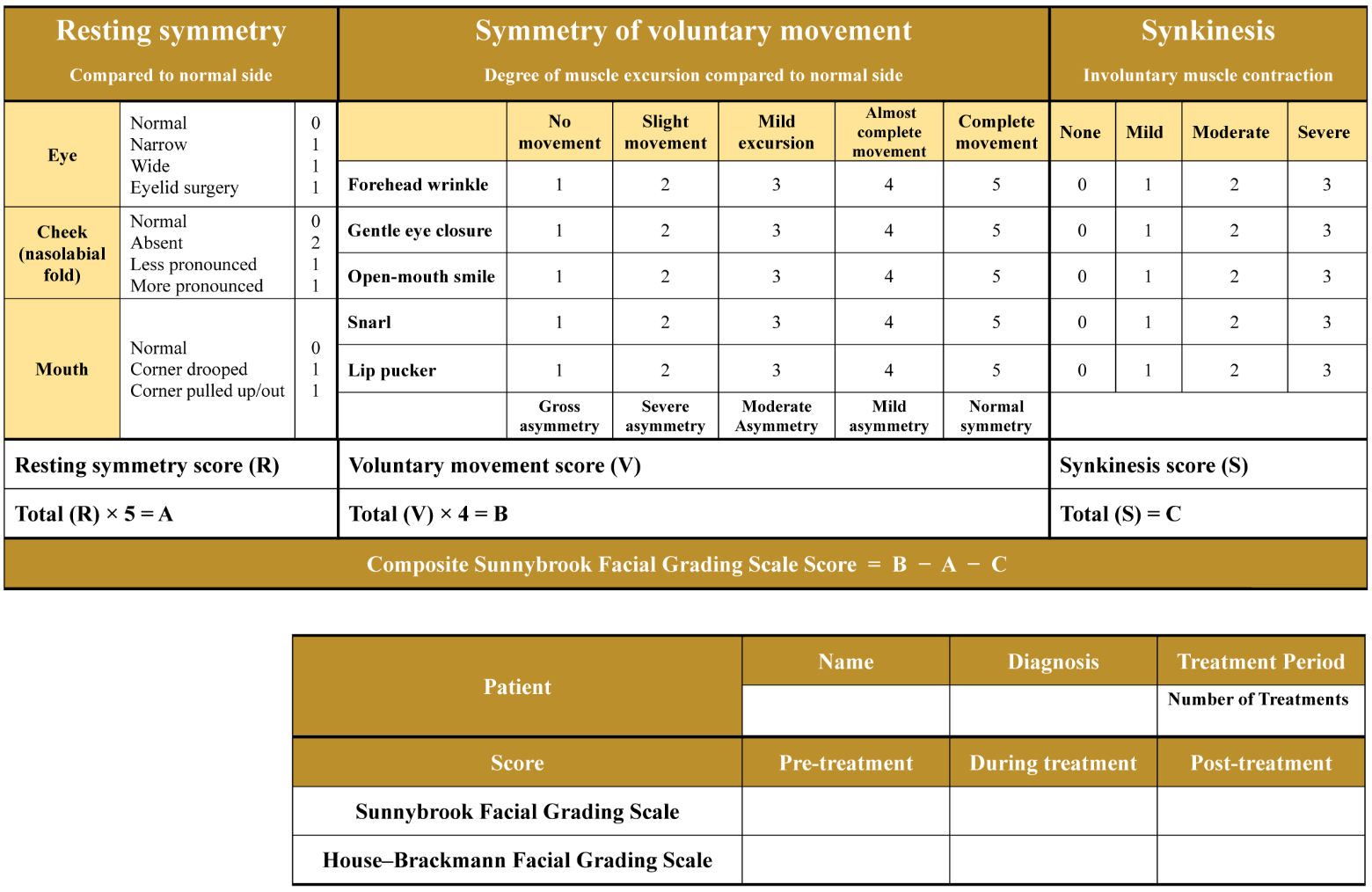
**

**Supplementary Figure 1. Patient Data Sheet for subjective composite scores for the** **House–Brackmann and Sunnybrook Facial Grading Scales.**
